# Supplementary material for: Impact of Gut Microbiome Modulation on Uremic Toxin Reduction in Chronic Kidney Disease: A Systematic Review and Network Meta-Analysis
Source: Nutrients. 2025 Apr 3;17(7):1247. doi: 10.3390/nu17071247 (PMC11990722; doi:10.3390/nu17071247)

Supplementary Materials

Supplemental material Figure S1. Individual study results (for all studies) grouped by treatment comparison

Urea

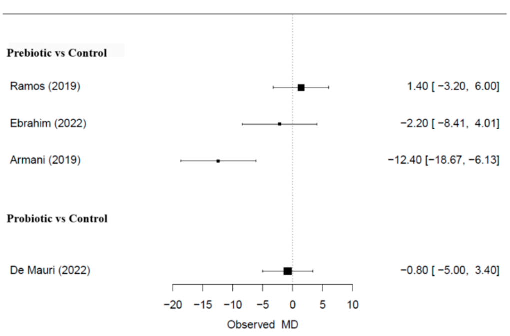

Creatinine

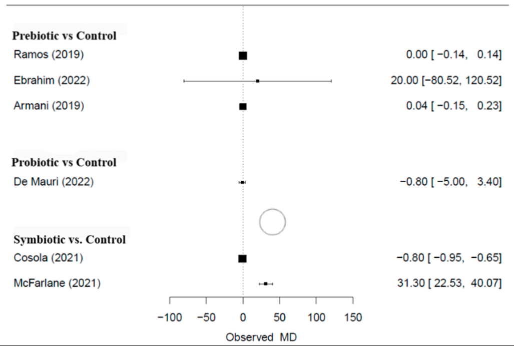

p-Cresyl Sulfate

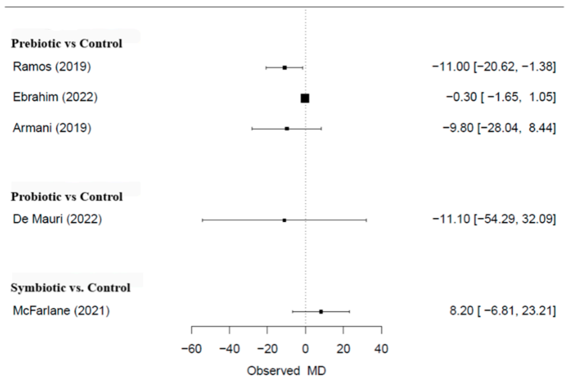

Free p-Cresyl Sulfate

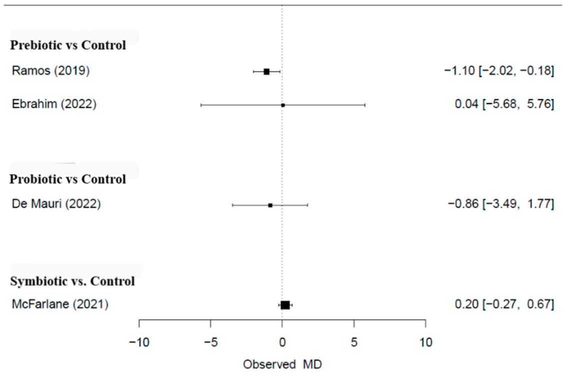

Total Indoxyl Sulfate

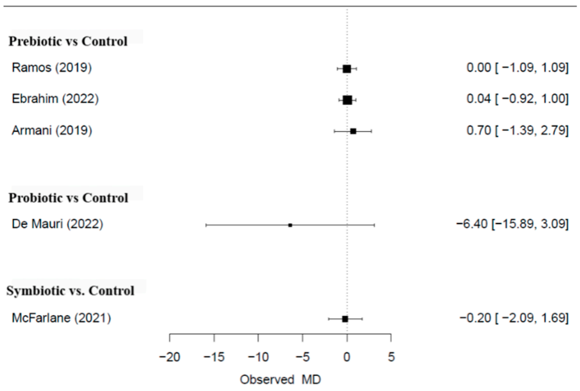

Free Indoxyl Sulfate

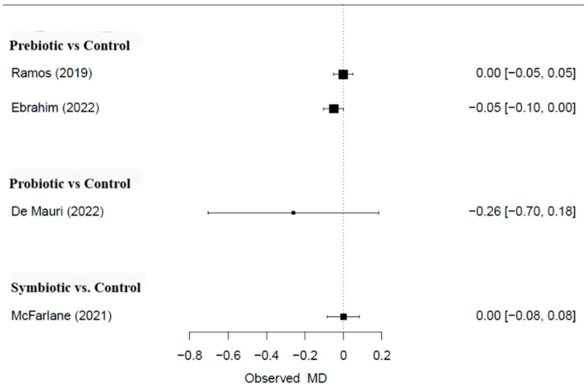

Supplement: Supplementary file 1 [file nutrients-17-01247-s001.zip › nutrients-3534718-supplementary.pdf]
